# Coordination of DNA repair by NEIL1 and PARP-1: a possible link to aging

**DOI:** 10.18632/aging.100492

**Published:** 2012-10-26

**Authors:** Nicole Noren Hooten, Megan Fitzpatrick, Kari Kompaniez, Kimberly D. Jacob, Brittany R. Moore, Julia Nagle, Janice Barnes, Althaf Lohani, Michele K. Evans

**Affiliations:** ^1^ Laboratory of Population Sciences, National Institute on Aging, National Institutes of Health, Baltimore, MD 21224; ^2^ Clinical Research Branch, National Institute on Aging, National Institutes of Health, Baltimore, MD 21224

**Keywords:** PARP, base excision repair, DNA damage, oxidative stress, aging, glycosylase

## Abstract

Oxidative DNA damage accumulates with age and is repaired primarily via the base excision repair (BER) pathway. This process is initiated by DNA glycosylases, which remove damaged bases in a substrate-specific manner. The DNA glycosylases human 8-oxoguanine-DNA glycosylase (OGG1) and NEIL1, a mammalian homolog of *Escherichia coli* endonuclease VIII, have overlapping yet distinct substrate specificity. Recently, we reported that OGG1 binds to the Poly(ADP-ribose) polymerase 1 (PARP-1), a DNA damage sensor protein that poly(ADP-ribosyl)ates nuclear proteins in response to DNA damage and other cellular signals. Here, we show that NEIL1 and PARP-1 bind both *in vitro* and *in vivo*. PARP-1 binds to the C-terminal-100 amino acids of NEIL1 and NEIL1 binds to the BRCT domain of PARP-1. NEIL1 stimulates the poly(ADP-ribosyl)ation activity of PARP-1. Furthermore, NEIL-deficient fibroblasts have impaired poly(ADP-ribosyl)ation of cellular proteins after DNA damage, which can be rescued by NEIL1 expression. Additionally, PARP-1 inhibits NEIL1 incision activity in a concentration-dependent manner. Consistent with the idea of impaired DNA repair during aging, we observed differential binding of PARP-1 to recombinant NEIL1 in older mice compared to younger mice. These data further support the idea that dynamic interplay between different base excision repair proteins is important for efficient BER.

## INTRODUCTION

Reactive oxygen species (ROS), including peroxides and free radicals, represent a continuous challenge for eukaryotic cells. ROS are generated endogenously as byproducts of oxidative phosphorylation during cellular respiration or exogenously by UV light, ionizing radiation, metal ions, chemotherapeutic agents, cigarette smoke and pesticides [1, 2]. These highly reactive molecules cause numerous DNA lesions including single-strand breaks (SSBs), double-strand breaks (DSBs), oxidized bases, cross links and abasic (apurinic/apyrimidinic; AP) sites [3, 4]. Failure to repair these lesions is thought to contribute to cancer, neurodegeneration and aging [5-7]. Accumulating evidence suggests that defects in DNA repair lead to increased oxidative DNA damage with age.

As a result, cells have evolved intricate mechanisms to effectively repair these lesions to preserve genomic stability and integrity. The highly conserved base excision repair (BER) pathway is primarily responsible for repairing oxidative DNA damage [8, 9]. DNA glycosylases initiate BER by locating and excising damaged bases. Monofunctional glycosylases hydrolyze N-glycosidic bonds leading to abasic sites. Glycosylases responsible for excising oxidized bases, however, are bifunctional, possessing intrinsic AP lyase activity and thus, also cleave the DNA backbone at the AP site [10-12]. AP endonuclease (APE1) or polynucleotide kinase (PNK) then removes the 3'-blocking group which generates a 3'OH terminus enabling a DNA polymerase to replace the excised nucleotide and a DNA ligase then seals the nick [9, 13]. Two broad groups of glycosylases, separated based on reaction mechanism, are responsible for removing oxidized bases: 8-oxoguanine-DNA glycosylase 1 (OGG1) and endonuclease III homolog (NTH1) make up the Nth family and catalyze β-elimination to form 5'-phosphate and 3'-phospho-α,β-unsaturated aldehyde termini and the more recently characterized endonuclease VIII-like 1, 2 and 3 (NEIL1, NEIL2, NEIL3) which make up the Nei/Fpg family and carry out βδ-elimination to generate 3'-phosphate and 5'-phosphate termini [10-12]. NEILs are unique in their ability to recognize and excise lesions on ssDNA, dsDNA and bubble DNA, whereas OGG1 and NTH1 require dsDNA [10-12]. NEIL1 is the best characterized of the Nei/Fpg family and is predominantly expressed during S-phase of the cell cycle, suggesting a possible role in the repair of DNA during replication and/or transcription [14]. Recent studies have demonstrated the importance of NEIL1 in maintaining genomic integrity as mutations in NEIL1 have been associated with gastric cancer and colorectal adenomas [15, 16]. Furthermore, NEIL1−/− mice develop metabolic syndrome, a condition characterized by obesity, dyslipidemia, fatty liver disease and hyperinsulinemia [17, 18]. Interestingly, this phenotype is specific for NEIL1−/− mice, as knockout of other glycosylases such as OGG1, MYH, and NTH display cancer susceptibility, but not metabolic syndrome [9], suggesting that the NEIL1 glycosylase may have unique repair and/or functional properties.

Binding of NEIL1 to interacting partners could influence the repair ability and localization of NEIL1. Protein interactions occur frequently during BER in order to coordinate steps and enhance efficacy of repair. NEIL1 has been shown to bind to a number of proteins involved in downstream BER steps including flap endonuclease 1 (FEN-1), proliferating cell nuclear antigen (PCNA), PNK, WRN, RPA, Rad9-Rad1-Hus1 complex, DNA polymerase β (Polβ) and DNA Ligase IIIα (LigIIIα) [19-24]. This suggests that NEIL1 binding to BER associated proteins is important in driving and regulating subsequent repair steps and supports the cellular interactome theory, which proposes that coordinated interactions between repair proteins are essential for the proper removal of DNA lesions.

Recently, we found that OGG1 binds to the ubiquitous DNA damage sensor protein Poly(ADP-ribose) polymerase (PARP-1) [25]. PARP-1 catalyzes the attachment of poly(ADP-ribose) (PAR) polymers to itself as well as histones, nuclear proteins, DNA repair proteins, transcription factors and chromatin modulators using NAD+ as a donor of ADP-ribose units [26-28]. Numerous studies have shown that PARP-1 activity is increased significantly by DNA damage, and previously we reported that OGG1 can also stimulate PARP-1 activity. Poly(ADP-ribosyl)ation after DNA damage is important for the recruitment of other damage response factors essential for efficient BER [26-28]. The important cellular role for PARP-1 is underscored by its critical role in inflammation and cancer [27, 28]. Furthermore, PARP-1 activity and/or expression may contribute to aging as mice lacking or overexpressing PARP-1 have changes in lifespan and differences in PAR levels have been observed with human age [26, 29-32].

Cells must combat constant threats from a large and diverse variety of oxidized bases, however, there are only a few glycosylases encoded in the human genome. As a result, glycosylases usually have broad, overlapping substrate specificity [5]. For example, although OGG1 prefers to remove 8-hydroxyguanine (8-oxoG) lesions, it can also excise formamidoguanine (FapyG), normally preferred by NEIL1 [33, 34]. This redundancy suggests that glycosylases can substitute for one another if necessary and implies that these molecules participate in common mechanisms to carry out BER. We show in this report that NEIL1, like OGG1, directly binds to and activates PARP-1 and that in turn, PARP-1 inhibits NEIL1 incision activity. In support of a functional interaction, cells deficient in NEIL1 show significantly decreased poly(ADP-ribosyl)ation after DNA damage. We also found that this complex is modulated in a physiological setting, as reduced binding of recombinant NEIL1 to PARP-1 was observed in old mice compared to young mice. This is interesting given the well-known role for DNA repair in aging. Taken together, these results provide evidence that the stable interaction of NEIL1 and PARP-1 plays an important role in oxidative DNA damage repair and that the interaction of PARP-1 with glycosylases may be a common mechanism to coordinate the repair process.

## RESULTS

### Association of NEIL1 with PARP-1 *in vitro* and *in vivo*

**Figure 1:**
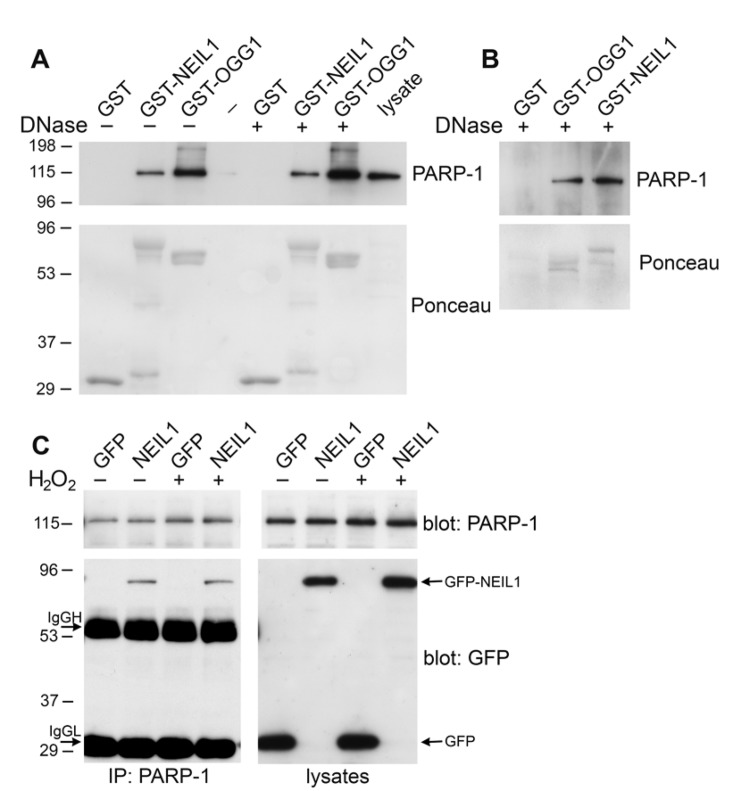
NEIL1 binds to PARP-1 (**A**) HeLa cells were lysed and incubated with GST, GST-OGG1 or GST-NEIL1 (20 μg) and then were either treated with DNase I (+) or mock treated (−). Samples were analyzed on a polyacrylamide gel, stained with Ponceau S and probed with anti-PARP-1 antibodies. ~1.5% of total PARP-1 bound to GST-NEIL1 and ~4% of total PARP-1 bound to GST-OGG1. (**B**) GST, GST-OGG1 and GST-NEIL1 (1 μg) were incubated with 250 ng purified PARP-1 *in vitro*. Samples were treated with DNase I and immunoblotted with anti-PARP-1 and anti-GST antibodies. ~5% of purified PARP-1 bound to both GST-OGG1 and GST-NEIL1. (**C**) HeLa cells were transfected with pEGFP or pEGFP-NEIL1 and mock treated (−) or treated (+) with 500 μM H_2_O_2_. PARP-1 immunoprecipitates were probed with anti-PARP-1 and anti-GFP antibodies. Arrows indicate GFP-NEIL1, immunoglobulin heavy chain (IgGH) and immunoglobulin light chain (IgGL) in the immunoprecipitates and GFP and GFP-NEIL1 in the lysates. ~1.5% of total PARP-1 was immuno-precipitated with GFP-NEIL1. There was no significant change in GFP-NEIL1 binding to PARP-1 after H_2_O_2_ treatment (P=0.25, n=3). Representative immunoblots are shown for each experiment and repeated in at least 3 independent experiments.

In order to examine whether PARP-1 binds to the NEIL1 DNA glycosylase, we initially purified wild-type NEIL1 fused to GST. We used this recombinant protein for GST precipitations from HeLa cell lysates. Immunoblotting these GST precipitations with PARP-1 antibodies revealed that GST-NEIL1 bound to PARP-1 similarly to GST-OGG1 (Figure 1A). To determine whether this interaction is direct, we incubated the GST-fusion proteins with purified PARP-1. Using this *in vitro* binding assay, we determined that PARP-1 binds directly to NEIL1 (Figure 1B). To exclude the possibility that the PARP-1/NEIL1 interaction occurs through DNA contamination in the purified proteins, we treated both precipitations from HeLa cells and from the *in vitro* binding assay with DNase I (Figure 1A,B). This interaction was observed despite DNase I treatment, suggesting that NEIL1/PARP-1 complex formation was mediated through a protein-protein interaction and not through DNA. In order to address whether PARP-1 binds to NEIL1 *in vivo*, we transfected HeLa cells with either GFP-NEIL1 or GFP control. GFP-NEIL1 coimmunoprecipitated with PARP-1 both in the presence and absence of DNA damage (Figure 1C). Previously, we found that binding of OGG1 to PARP-1 was enhanced by treatment with the DNA damaging agent H_2_O_2_ [25]. However, we did not observe a difference in the stable interaction between NEIL1 and PARP-1 in response to oxidative stress (Figure 1C). In support of this finding, we have found *in vitro* that purified NEIL1 can bind to both PARP-1 and auto(ADP-ribosyl)ated PARP-1 (data not shown).

### Mapping the binding regions of NEIL1 and PARP-1

**Figure 2:**
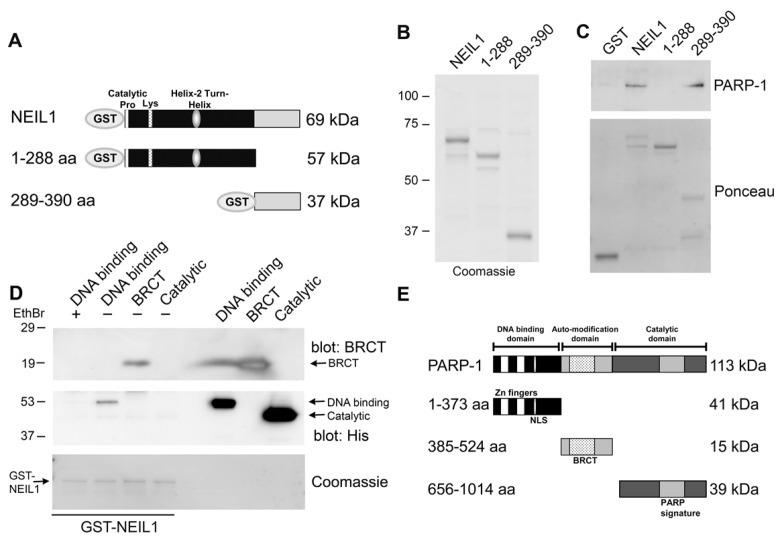
Mapping the interacting regions of PARP-1 and NEIL1 (**A**) Schematic representation of GST-NEIL1 fusion proteins. (**B**) Purified GST-NEIL1 fusion proteins were analyzed by SDS-PAGE followed by staining with Coomassie Colloidal Blue. (**C**) PARP-1 binds to the C-terminal 289–390 aa of NEIL1. PARP-1 (250 ng) was incubated with either GST, GST tagged full-length NEIL1 or indicated NEIL1 fragments (1 μg) in an *in-vitro* binding assay. GST precipitations were probed with anti-PARP-1 antibodies and stained with Ponceau S. ~5% of purified PARP-1 bound to both GST-NEIL and NEIL 289–390aa. (**D**) NEIL1 binds to the BRCT domain of PARP-1. The DNA binding (41 kDa), BRCT (15 kDa) and catalytic (39 kDa) domains of PARP-1 (1 μg) were incubated with 1 μg GST-NEIL1 (69 kDa) either in the presence (+) or absence (−) of ethidium bromide. GST-NEIL1 precipitations were immunoblotted with anti-His (for the DNA binding and catalytic domains) and anti-BRCT (for BRCT domain) antibodies and then stained with Coomassie to reveal the amount of GST-NEIL1 in the precipitations. The different PARP-1 domains and GST-NEIL1 are indicated by arrows. ~4% of purified BRCT domain bound to GST-NEIL1. (**E**) A schematic of the different PARP-1 proteins is shown.

To gain further insight about this interaction, we purified two fragments of NEIL1 corresponding to the first 1–289 aa and the C-terminal 289–390 aa (Figure 2A,B). Colloidal Coomassie Blue staining of the polyacrylamide gel allowed visualization of the purified fragments (Figure 2B). PARP-1 bound to the C-terminal 289–390 aa of NEIL1 (Figure 2C) in the *in vitro* binding assay. Interestingly, other proteins including, WRN, FEN-1, PCNA, RPA, Rad9-Rad1-Hus1 complex, DNA polymerase β, and DNA ligase IIIα have also been shown to bind to NEIL1 in this region, suggesting that this domain may be important for mediating protein-protein interactions [19-24]. In addition, we examined whether two single nucleotide polymorphisms of NEIL1 in this region (R339Q, R334G) affected PARP-1 binding [15, 16]. However, both of these cancer-associated polymorphic variants retained the ability to bind to PARP-1 (data not shown). Additionally, we mapped the region of PARP-1 where NEIL1 binds (Figure 2D) by incubating GST-NEIL1 with either the DNA binding, BRCT or catalytic domains of PARP-1 (Figure 2E). We immunoblotted these GST-NEIL precipitations with either anti-His antibodies that recognize the DNA binding and catalytic domains or antibodies specific for the BRCT domain. NEIL1 bound to the BRCT domain of PARP-1, a region important for protein binding (Figure 2D). We also found that NEIL1 bound to the PARP-1 DNA binding domain, but this binding was abrogated by incubation with the DNA intercalator ethidium bromide, indicating that this interaction is mediated mainly through DNA. These data suggest that NEIL1 binds to PARP-1 through a protein-protein interaction with the BRCT domain of PARP-1.

### Inhibition of NEIL1 incision activity by PARP-1

**Figure 3:**
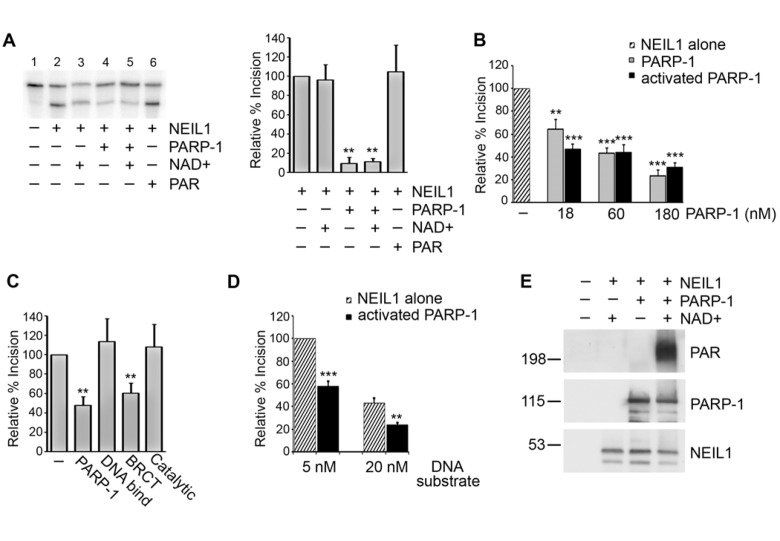
PARP-1 inhibits NEIL1 incision activity (**A**) NEIL1 (38 nM; 33 ng) was incubated with buffer, NAD+ with or without PARP-1 (180 nM; 400 ng), or with PAR and reacted with a 5'- ^32^P-labeled oligonucleotide duplex containing a 5OHU lesion for 15 min at 37°C. The cleavage products were analyzed on a 20% denaturing gel containing 7 M urea. Percent incision was calculated by normalizing the amount of cleaved substrate (bottom band) to the amount of uncleaved product (top band). Data was then normalized to the amount of incision activity of NEIL1 alone (100%). (**B**) Incision assays were performed as in (**A**) in the presence of the indicated concentrations of PARP-1 with (activated PARP-1) or without NAD+ (PARP-1) and quantified. (**C**) NEIL1 (33 ng) incision assays were performed in the presence of 400 ng of the indicated PARP-1 domains or full-length PARP-1 in the presence of NAD+ (10 μM). (**D**) PARP-1 inhibits NEIL1 activity at both 5 nM and 20 nM concentrations of DNA substrate. The histograms in (**A**) and (**B**) represent the mean + SEM from three, (**C**) from five, and (**D**) from four independent experiments.**p≤0.01 and ***p≤0.001 compared to the incision activity of NEIL1 alone using one-way ANOVA and Tukey's post-hoc test. (**C**) NEIL1 was incubated with buffer or PARP-1 with or without NAD+ and incubated with a non-radiolabeled oligonucleotide containing the 5OHU lesion as above. Samples were separated by SDS-PAGE and probed with anti-PAR, anti-PARP-1 and anti-NEIL1 antibodies.

To determine whether the binding of PARP-1 to NEIL1 has functional consequences, we first examined the ability of NEIL1 to excise a 5-OHU lesion from a radiolabeled duplex oligonucleotide substrate. NEIL1 effectively excised the 5-OHU lesion in the absence of PARP-1 (Figure 3A, Lane 2). In contrast, when PARP-1 was present NEIL1 activity was significantly inhibited (Figure 3A, Lanes 4,5). PARP-1 requires the substrate NAD+ for the synthesis and attachment of PAR. We were interested in whether PARP-1 differentially inhibits NEIL1 depending on its activation and auto(ADP-ribosyl)ation status. Interestingly, PARP-1 inhibited NEIL1 activity both in the presence and absence of the necessary cofactor NAD+ in a concentration-dependent manner, suggesting that PARP-1 decreases NEIL1 activity regardless of activation and modification (Figure 3A,B, Lanes 4,5). Furthermore, the cofactor NAD+ alone did not affect NEIL1 activity (Figure 3A, Lane 3), suggesting that the observed effects are specific to PARP-1. In these experiments, a molar excess of PARP-1 was used, but inhibition of NEIL1 activity was observed over a range of concentrations of PARP-1 including submolar concentrations of PARP-1 (Figure 3B). Addition of PAR to the reaction did not substantially affect NEIL1 incision activity (Figure 3A, Lane 6), suggesting that inhibition of NEIL1 activity is dependent on PARP-1. In support of this idea, the BRCT domain of PARP-1, which binds to NEIL1, also inhibits NEIL1 incision (Figure 3C). In contrast, the DNA binding domain and catalytic domain, which do not bind NEIL1, do not affect NEIL1 activity (Figure 3C). Similar levels of PARP-1 inhibition on NEIL1 activity were observed at increased amounts of DNA substrate, further indicating that protein-protein interactions are responsible for the observed effects (Figure 3D).

To ensure that PARP-1 is activated in the incision assays, we incubated NEIL1 and PARP-1 with or without NAD+ and then added the duplex oligonucleotide containing the 5-OHU lesion as in the incision assays. However, in these experiments the duplex 5-OHU substrate was not radiolabeled. After incubation, we separated the reactions on a poly-acrylamide gel and immunoblotted with anti-PAR antibodies to monitor PARP-1 activity (Figure 3E). Indeed, in the presence of NAD+, PARP-1 was substantially activated in the incision assays, indicating that the similar inhibitory actions of PARP-1 and activated PARP-1 on NEIL1 incision ability were not due to the fact that PARP-1 was not active in our experiments.

### Enhancement of PARP-1 activity by NEIL1

**Figure 4:**
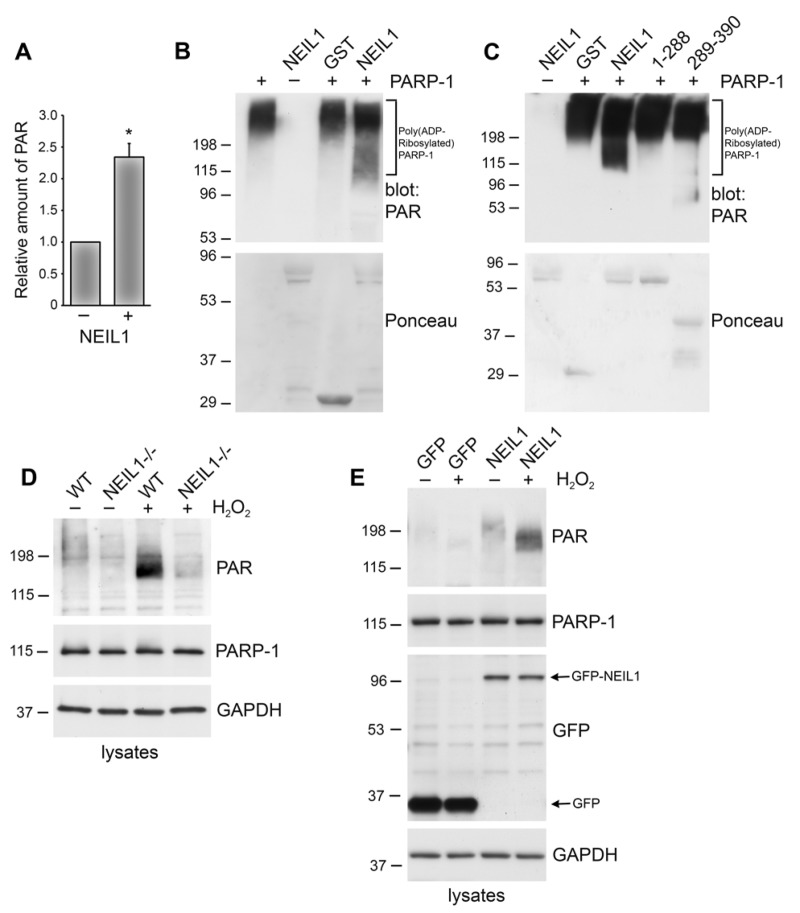
NEIL1 stimulates the poly(ADP-ribosyl)ation activity of PARP-1 (**A**) PARP-1 activity was measured by determining the amount of PAR deposited on immobilized histones in an ELISA assay. Addition of NEIL1 (33 ng) increased the amount of PAR synthesis by PARP-1 (2 ng). The histogram represents the mean + SEM from three independent experiments. *p < 0.05 compared to control by Student's t-test. (**B,C**) GST, GST-NEIL1, GST-NEIL1 1–288 aa or GST-NEIL1 289–390 aa (5 μg) were incubated with (+) or without (−) 7.5 ng PARP-1. The immunoblot was stained with Ponceau S and probed with anti-PAR antibodies. (**D**) WT or NEIL1−/− MEFs were mock treated (−) or treated (+) with 500 μM H_2_O_2_. Lysates were probed with anti-PAR antibodies and reprobed with anti-PARP-1 and anti-GAPDH antibodies as a protein loading control. (E) NEIL1−/− MEFs transfected with pEGFP-NEIL1 or pEGFP control were mock treated (−) or treated (+) with 500 μM H_2_O_2_ and lysates were probed as above. Anti-GFP antibodies were used to examine levels of the transfected proteins. For (**B-E**), a representative experiment from at least 3 independent experiments is shown.

Stimulation of the poly(ADP-ribosyl)ation activity of PARP-1 plays an important role in the repair of oxidative DNA damage. Therefore, we wanted to examine whether NEIL1 effects the poly(ADP-ribosyl)ation activity of PARP-1. Initially, we performed an ELISA assay that measures the amount of PAR deposited by PARP-1 on immobilized histones and found that addition of NEIL1 increased the poly(ADP-ribosyl)ation of histones by PARP-1 (Figure 4A). To test whether NEIL1 also stimulates PARP-1 automodification, we performed *in vitro* ribosylation reactions with purified proteins. GST-NEIL1 or GST control were preincubated with PARP-1, followed by the addition of activated DNA and NAD+. The reactions were separated on a polyacrylamide gel and the accumulation of PAR was visualized by immunoblotting with anti-PAR specific antibodies. PARP-1 activity was not affected by incubation with GST control (Figure 4B). However, GST-NEIL1 enhanced PARP-1 activity above background level of PARP-1 activation (GST control lane) (Figure 4B). We also incubated the two NEIL1 fragments with PARP-1. Incubation with GST-NEIL1 289–390 aa, the domain that binds PARP-1, slightly increased PARP-1 activity but not to the level of activation observed with wild-type NEIL1. Furthermore, the 1–288 aa fragment did not activate PARP-1 above background GST levels (Figure 4C). These data indicate that full-length NEIL1 is necessary for enhanced PARP-1 activation. In these reactions, we did not observe a shift in the mobility of GST-NEIL1, an indication of poly(ADP-ribosyl)ation, nor was there any detectable PAR signal around the molecular weight of GST-NEIL1, indicating that NEIL1 is not substantially poly(ADP-ribosyl)ated by PARP-1 (data not shown). In support of this idea, in our immunoprecipitation experiments we did not detect a PAR signal at the molecular weight of GFP-NEIL1, suggesting that NEIL1 is not significantly poly(ADP-ribosyl)ated *in vivo* (data not shown).

In addition to NEIL1 activating PARP-1 *in vitro*, we also wanted to examine whether NEIL1 affects PARP-1 activity in cells. NEIL1 could affect poly(ADP-ribosyl)ation levels in cells by either affecting PARP-1 activity or PARP-1 expression. To address these different possibilities, we first examined PARP-1 levels in lysates of cells either expressing NEIL1 or lacking NEIL1 expression (Figure 4D). We did not observe a significant difference in PARP-1 expression in mouse embryo fibroblasts (MEFs) lacking NEIL1 compared to wild-type (Figure 4D).

To further investigate whether NEIL1 affects PARP-1 activity in cells, we modulated NEIL1 expression and then examined the poly(ADP-ribosyl)ation of cellular proteins in response to DNA damage. MEFs that were lacking NEIL1 or WT control were mock treated or treated with the DNA damaging agent, H_2_O_2_, which has previously been shown to potently activate PARP-1 [25, 35]. Cell lysates were separated on polyacrylamide gel and immunblotted with PAR antibodies. As expected, a low level of PAR reactivity was observed in untreated cells. In contrast, H_2_O_2_ treatment increased the poly(ADP-ribosyl)ation of cellular proteins in WT cells (Figure 4D). Typical for this posttranslational modification, the major PAR signal was detected as a high molecular weight smear, and most likely corresponds to auto(ADP-ribosyl)ated PARP (see Figure 4B). NEIL1−/− MEFs had greatly diminished poly(ADP-ribosyl)ation levels after exposure to H_2_O_2_ compared to WT cells (Figure 4D). To test whether expression of NEIL1 could rescue the poly(ADP-ribosyl)ation defect in cells lacking NEIL1, we transfected NEIL1−/− MEFs with GFP-NEIL1 or GFP control (Figure 4E). GFP-NEIL1 restored PAR levels similar to those observed in WT cells after H_2_O_2_ treatment, whereas low levels of PAR were observed after H_2_O_2_ treatment of GFP-expressing MEFs. These data indicate that NEIL1 stimulates PAR polymer formation both *in vitro* and *in vivo*.

### Decreased binding of GST-NEIL1 to PARP-1 in older mice

**Figure 5:**
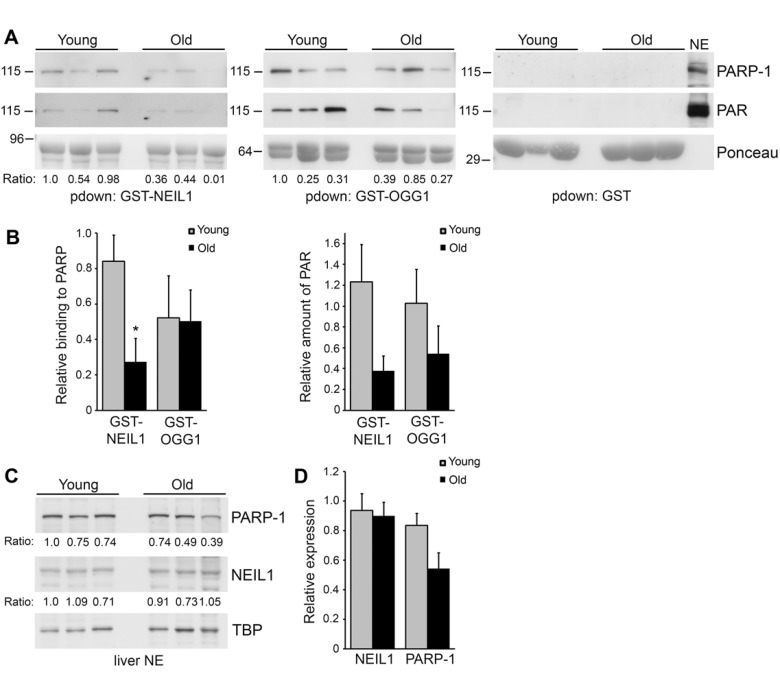
Decreased binding of PARP-1 to GST-NEIL1 with mouse age (**A**) Liver nuclear extracts were made from 3 different young (7 month) and 3 different old (17 month) mice and used for GST-precipitations with the indicated fusion proteins. Precipitations were probed with anti-PARP-1 and anti-PAR antibodies and stained with Ponceau S to visualize the fusion proteins. The relative amount of PARP-1 binding and PAR levels in precipitations were quantified from immunoblots and normalized to the amount of GST-fusion protein. The numbers below the blots represent the relative level of PARP-1 binding and the histograms in (**B**) represent the average PARP-1 binding and PAR levels in precipitations from the 3 young and 3 old mice. *P<0.05 comparing young and old mice using Student's t-test. Similar results were obtained in another independent experiment. (**C**) Liver nuclear extracts from young and old mice were probed by immunoblotting with anti-PARP-1 and anti-NEIL1 antibodies and reprobed with anti-TBP antibodies as a protein loading control. The numbers below the blots represent the level of PARP-1 or NEIL1 normalized to TBP. The histogram in (**D**) represents the average levels of NEIL1 and PARP-1 + SEM from the 3 young and 3 old mice.

Accumulation of DNA damage and defects in DNA repair are thought to contribute to aging and age-related diseases [36-38]. Specifically, changes in PARP activity have been observed in immortalized lymphocytes from young, old and centenarian individuals and changes in PARP-1 expression affect the lifespan of mice, indicating that PARP-1 may play a role in aging and longevity [29-32, 39]. Therefore, we wanted to examine whether PARP-1 binding to GST-NEIL1 changes with mouse age. We made liver nuclear extracts from 3 different young mice (7 months) and 3 different older mice (17 months) and performed GST-precipitations to examine PARP-1 binding. Interestingly, we found a significant reduction in binding of PARP-1 to GST-NEIL1 in old versus young mice (Figure 5A,B). This is not a general effect of PARP-1 binding as we found that GST-OGG1 bound to PARP-1 similarly regardless of mouse age and PARP-1 did not bind to GST control (Figure 5A,B). We observed that there was a decrease, although non-significant, in PARP-1 protein expression with age, indicating that reduced binding of PARP-1 to GST-NEIL1 may in part be due to lower levels of PARP-1 in older mice (Figure 5C,D). PARP-1 activity levels may also contribute to decreased binding of PARP-1 to NEIL1, as reduced levels of PAR were observed in GST-NEIL1 precipitations from old mice (Figure 5A,B).

## DISCUSSION

BER is a vital cellular mechanism to repair DNA lesions induced by ROS. This process is thought to involve a network of pathways consisting of dynamic and highly coordinated protein interactions that work to increase efficiency and maintain genomic integrity and homeostasis [40, 41]. In this study, we identified a novel interaction between the DNA glycosylase NEIL1 and the DNA damage sensor protein PARP-1. We determined that NEIL1 binds directly to the BRCT domain of PARP-1, a region that often mediates interactions between proteins involved in the DNA damage response or cell cycle checkpoints such as OGG1, XRCC1, Polβ and histones [25, 42, 43]. Furthermore, we also observed that PARP-1 binds to residues 289–390 at the C-terminus of NEIL1. This region acts as a common interface for the binding of several proteins involved in downstream steps of BER including FEN-1, Polβ, LigIIIα and PCNA [20, 21, 24]. The observation that PARP-1 also binds to this region provides further support for the idea that DNA glycosylases, such as NEIL1, act as hub proteins by interacting with numerous factors involved in BER in order to drive downstream steps. This region of NEIL1 has no sequence similarity to the PARP-1 binding domain in OGG1 (79–180aa)[25]. Structurally this region in OGG1 consists of five α helixes andtwo β-sheets, whereas in NEIL1 the area where PARP-1 binds has been hypothesized to be disordered based on software analysis and the lack of obvious structure in this region using crystallography [40, 44, 45]. It is also interesting to note that this region is absent in the bacterial prototype of NEIL1, Nei, which insinuates that through evolution NEIL1 has acquired this binding domain for mediating multi-protein interactions in order to facilitate BER and combat the increased levels of oxidative stress that higher organisms face [40].

Interestingly, PARP-1 inhibited the ability of NEIL1 to excise DNA lesions regardless of whether or not the required PARP-1 cofactor NAD+ was present. In agreement with this observation, we found that NEIL1 interacted with auto(ADP-ribosyl)ated as well as unmodified PARP-1 (data not shown), and our co-immunoprecipitation experiments indicated that the stable interaction between NEIL1 and PARP-1 was not altered by oxidative stress. This contrasts with our previous work where we found that OGG1 was only inhibited by activated PARP-1 and that the OGG1/PARP-1 complex formation was enhanced by DNA damage [25]. Intriguingly, many of the characterized binding partners of NEIL1 such as WRN, FEN-1, Rad9-Rad1-Hus1 complex and PCNA stimulate incision activity [19-21, 23]. It appears that we have uncovered a common inhibitory effect that PARP-1 has on glycosylase activity. It will be interesting in the future to examine whether PARP-1 interacts and possibly inhibits the activity of other glycosylases.

We used several experimental approaches to demonstrate that NEIL1 activates PARP-1. First, *in vitro* poly(ADP-ribosyl)ation assays showed that NEIL1 stimulated PARP-1 automodification. Secondly, NEIL1 enhanced the poly(ADP-ribosyl)ation of histones by PARP-1. Thirdly, cells lacking NEIL1 were defective in the poly(ADP-ribosyl)ation of cellular proteins in response to DNA damage. Fourthly, expression of NEIL1 in NEIL1−/− MEFs increased the poly(ADP-ribosyl)ation of cellular proteins after DNA damage, but did not significantly change PARP-1 expression. However, we cannot exclude the possibility that the activity or expression of other repair factors may be altered by NEIL1 expression and contribute to the poly(ADP-ribosyl)ation pathway or that NEIL1 may also affect other PARP isoforms.

Based on our results, it is possible that NEIL1 and PARP-1 form a constitutively bound multiprotein complex that continuously monitors the DNA for breaks and damaged bases. Alternatively, NEIL1 excision of the damaged base could recruit PARP-1 to the site where it can bind to NEIL1. NEIL1 may then stimulate PARP-1 automodification and poly(ADP-ribosyl)ation of other nuclear proteins and histones. Poly(ADP-ribosyl)ation by PARP-1 plays an important role in DNA repair by activating and recruiting other proteins to the DNA strand break such as the scaffolding protein XRCC1, which is responsible for activation of the majority of the repair enzymes and has also been shown to associate with NEIL1 using purified proteins [43, 46, 47]. Therefore, it is conceivable that NEIL1 activation of PARP-1 enhances the formation of a multiprotein complex that could include other BER factors such as XRCC1. NEIL1 may also enhance PARP-1 activity by creating abasic sites that would be converted by PNK to SSBs that could further potentiate PARP-1 activity [24]. PARP-1 may then inhibit incision activity of NEIL1, possibly to enable the enzyme to better interact with other downstream repair enzymes or to remove NEIL1 from the lesion in order to allow for subsequent BER steps.

This complex could play a role in repairing endogenous or induced DNA damage as well as replication-associated repair. NEIL1 has been implicated in BER associated with replication based on its upregulation during S-phase, specific recognition of fork structures and association with factors of the replication machinery [14, 40, 48]. XRCC1 and PARP-1-containing complexes have also been implicated in replication-associated repair, a process that is critical for preventing the incorporation of damaged bases into the DNA. Perhaps this unique role for the NEIL1 glycosylase may in part explain the more pronounced phenotypic defects of NEIL1−/− mice compared to other single glycosylase knockouts [9, 11, 17]. Consistent with this, we have found that colony formation is impaired in NEIL1−/− MEFs compared to wild-type, whereas, there was little or no difference between OGG1−/− MEFs and their wild-type counterparts in the absence of DNA damage [25, data not shown]. Future work lies in defining the role of NEIL1 in different repair complexes as they may depend upon the level or type of DNA damage, as well as, the stage of the cell cycle [49].

The functional consequences of the interaction between NEIL1 and PARP-1 may also be important in aging phenotypes. PARP-1 has been hypothesized to play a role in aging. Previous studies have shown that high PARP activity is associated with longer lived species [39]. The role of PARP-1 in aging is complex due to its dual-role as a protector of genomic integrity and promoter of inflammation. In support of this idea, both mice lacking PARP-1 and mice ectopically overexpressing human PARP-1 die prematurely from age-related pathologies [29, 32]. These studies suggest that tight regulation of PARP-1 expression is essential to maintain homeostasis. In our study, we found significantly reduced precipitation of PARP-1 with GST-NEIL1 in older mice compared to young mice. This decreased binding could be due to decreased PARP-1 expression, differential levels of PARP-1 activity and/or accessibility of PARP-1. We found that PARP-1 expression is non-significantly lower in older mice versus younger mice, which suggests that decreased expression alone likely does not account for the diminished binding of PARP-1 to GST-NEIL1 with mouse age. In support of this idea, PARP-1 activity, rather than expression, has been reported to be higher in centenarians then the general population [31] and we found decreased levels of PAR in the GST-NEIL1 precipitations from old mice. However, one report showed increased PARP-1 levels in centenarians versus older individuals, but this was observed in a limited number of samples [30]. Future work lies in determining the relationship between PARP-1 expression and activity in aging and longevity.

These data suggest that direct binding between the DNA glycosylase NEIL1 and the DNA damage sensor protein PARP-1 may help coordinate the repair of DNA damage. As PARP-1 inhibitors are currently being used clinically as anti-cancer therapies, characterizing the cellular role of PARP-1 is crucial for understanding the therapeutic potential of these agents [50, 51]. Furthermore, as defects in DNA repair occur with age, it is important to decipher the mechanisms that drive repair processes in order to further understand the contribution of these pathways to aging and age-related diseases.

## METHODS

### Cell Culture and Transfections

HeLa cells were cultured in Dulbecco's modified Eagle's medium (DMEM) containing 10% fetal bovine serum (FBS). WT and NEIL1−/− mouse embryo fibroblasts (MEFs) were a gift from R. Stephen Lloyd's laboratory (Oregon Health and Science University) and cultured in DMEM supplemented with 10% FBS. The pCMV-2B vector was from Stratagene. pEGFP-C1-NEIL1 was generated by PCR using pCMV6-XL5-NEIL1 (OriGene Technologies) as a template and verified by sequencing. HeLa and NEIL1−/− cells were transfected with Lipofectamine 2000™ according to manufacturer's instructions and cells were harvested 24 hrs after transfection.

pGEX4T2 constructs containing NEIL1, NEIL1 1–288 aa or NEIL1 289–390 aa were generated by PCR using pCMV-2B NEIL1 as a template. The PCR products were digested and ligated into the pGEX4T2 vector using the EcoR1 and Xho1 sites. Plasmids were verified by sequencing.

### GST purification and precipitations

GST proteins were purified using standard procedures and precipitations were performed essentially as previously described [25]. For Figure 1A, 20 μg GST, GST-NEIL1 or GST-OGG1 were incubated with HeLa cell lysate for 1 hr at 4°C and subsequently mock treated or treated with 10 units of DNase I (RNase-free, Ambion) for 15 min at 37°C. Precipitations were extensively washed to remove unbound proteins and resolved by SDS-PAGE followed by immunoblotting with anti-PARP-1 (Cell Signaling), or anti-GST (Santa Cruz) antibodies. Membranes were also stained with Ponceau S to visualize GST fusion proteins.

For *in vitro* binding assays, recombinant high purity PARP-1 (250 ng; Alexis Biochemicals) was incubated with 1 μg GST or GST-NEIL1 for 1 hr at 4°C and then DNase I treated as above. In Figure 2B, NEIL1 fragments (1–288 aa or 289–390 aa) or NEIL1 were separated by SDS-PAGE and the gel was stained with Colloidal Coomassie Blue. These same fusion proteins (1 μg) were incubated with 250 ng PARP-1 and processed as above. To map the region where NEIL1 binds to PARP-1, we incubated GST-NEIL1 (1 μg) with 1 μg of His-tagged proteins corresponding to the DNA binding domain (1–373 aa;[25]), BRCT domain (385–524 aa; Alexa Biochemicals) and catalytic domain (656–1014 aa; Alexa Biochemicals) of PARP-1. Experiments were performed as above. The DNA binding domain samples were either incubated in the absence or presence of 10 μg/ml ethidium bromide. The fusion proteins alone (100 ng) were loaded as positive controls. The samples were separated by SDS-PAGE and immunoblotted with anti-His polyclonal antibodies (HRP-conjugated; Abcam) or anti-PARP-1 antibodies against the BRCT domain (Calbiochem; 512737). Membranes were stained with either Ponceau S or Coomassie Blue to visualize the fusion proteins.

### Immunoprecipitations

HeLa cells transfected with either pEGFP or pEGFP-NEIL1 were mock treated or treated with 500 μM H_2_O_2_ for 30 min. Immunoprecipitations were performed essentially as previously described [52] and PARP-1 was immunoprecipiated with anti-PARP-1 antibodies from Enzo Life Sciences (clone C-2-10). PARP-1 immunoprecipitates or lysates were probed with anti-GFP (Millipore) and anti-PARP-1 antibodies (Cell Signaling).

### DNA Incision Activity Assays

A 30-mer oligo-nucleotide containing an 5-hydroxyuracil (5OHU) at position X-TTCTCTCTTTCCTTXTCTCTTTCTCTCTTC and the complementary oligonucleotide where G is opposite the X (both from Midland Certified Reagent Company, Midland, TX) were 5'-^32^P-labeled as previously described [53]. NEIL1 glycosylase/AP lyase activity was measured by incubating 38 nM untagged NEIL1 (NEB) with either 180 nM untagged PARP-1 (or the indicated concentrations), 10 μM NAD+, 0.01 nM PAR (all from Trevigen) in 12 μl reactions containing (20 mM Tris-HCl, pH 7.4, 100 mM NaCl, and 0.15 μg/μl BSA) for 10 min at 4°C then 8 μl radiolabeled oligonucleotides (5 nM) were added. The reactions were incubated at 37°C for 15 min and then stopped by the addition of loading buffer (90% deionized formamide, 1X Tris borate EDTA, 0.1% bromophenol blue, 0.1% xylene cyanol). Samples were heated at 95°C for 5 min and were run on 20% acrylamide gels containing 7 M urea. Radioactivity was measured using a Storm Phosphoimager and quantified using ImageQuant software (Molecular Dynamics). Percent incision was calculated by taking the amount of the lower cleaved band normalized to the amount of uncleaved substrate (top band). Background level of incision in buffer lane alone was subtracted from all lanes. The data was normalized to the incision activity of NEIL1 alone (100%).

The experiment in Figure 3C was performed as in the incision assays described above with the exception that the duplex oligonucleotides were not ^32^P-labeled. Also, the samples were stopped by the addition of 2X Laemmli buffer, separated on 10% polyacrylamide gels and probed by immunoblotting with anti-PAR antibodies (Trevigen) and reprobed with anti-PARP-1 (Cell Signaling) and anti-NEIL1 (Calbiochem) antibodies.

### Poly (ADP-ribose) PAR Assays

In order to examine whether NEIL1 affects PARP-1 activity, we used the HT Colorimetric PARP/Apoptosis Assay (Trevigen) that measures the amount of PAR deposited on immobilized histones. The assay was performed according to manufacturer's instructions with the exception that 33 ng NEIL1 (NEB) was incubated with the PARP-1-high specific activity enzyme (2 ng) for 10 min prior to the addition of the components of the poly(ADP-ribosyl)ation reaction (activated DNA and PARP-1 cocktail). The absorbance at 630 nm was measured using an ELISA plate reader. Measurements taken without PARP-1 enzyme were subtracted as background.

To measure PARP-1 automodification, we incubated PARP-1-HSA (Trevigen; 7.5 ng) with GST-NEIL1, GST-NEIL1 1–288 aa, GST-NEIL1 289–390 aa or GST control (5 μg) for 10 min and then added PARP cocktail, activated DNA and buffer (Trevigen), incubated for 15 min and then the reactions were separated by SDS-PAGE. Reactions were probed by immunoblotting with anti-PAR antibodies (Trevigen) and membranes were stained with Ponceau S to visualize the fusion proteins.

To detect PAR polymer formation by immunoblotting, wild-type and NEIL1−/− MEFs were untreated or treated with 500 mM H_2_O_2_ for 30 min and lysed in RIPA buffer. Lysates were separated by SDS-PAGE and immunoblotted with anti-PAR antibodies (Trevigen) and reprobed with anti-PARP-1 (Cell Signaling), anti-GFP (Millipore) and anti-GAPDH antibodies (Santa Cruz Biotechnology), as a protein loading control.

### GST precipitations from young and old mice

Frozen C57Bl/6 mouse livers from 3 different young (7 month) and 3 different older (17 month) mice were a gift from Dr. Rafael de Cabo (NIA). Livers were thawed and nuclear extracts were made as previously described [25]. Equal amounts of liver nuclear extracts (175 μg) were diluted into IP buffer [25] and incubated with GST, GST-NEIL1 or GST-OGG1 (20 μg) for 1 hr at 4°C. Precipitations were washed 5X with IP buffer and analyzed by SDS-PAGE followed by immunoblotting with anti-PARP-1 antibodies (Cell Signaling). Membranes were reprobed with anti-PAR antibodies (Trevigen) and stained with Ponceau S to examine loading of the GST fusion proteins. In addition, liver nuclear extracts (30 μg) were separated by SDS-PAGE and probed with anti-PARP-1 (Cell Signaling), anti-NEIL1 (Calbiochem) and anti-TBP antibodies (Abcam).

## References

[R1] Svilar D, Goellner EM, Almeida KH, Sobol RW (2011). Base excision repair and lesion-dependent subpathways for repair of oxidative DNA damage. Antioxidants & redox signaling.

[R2] Barnes DE, Lindahl T (2004). Repair and genetic consequences of endogenous DNA base damage in mammalian cells. Annual review of genetics.

[R3] Bjelland S, Seeberg E (2003). Mutagenicity, toxicity and repair of DNA base damage induced by oxidation. Mutat Res.

[R4] Dizdaroglu M, Jaruga P, Birincioglu M, Rodriguez H (2002). Free radical-induced damage to DNA: mechanisms and measurement. Free Radic Biol Med.

[R5] Hegde ML, Mantha AK, Hazra TK, Bhakat KK, Mitra S, Szczesny B (2012). Oxidative genome damage and its repair: Implications in aging and neurodegenerative diseases. Mechanisms of ageing and development.

[R6] Caldecott KW (2008). Single-strand break repair and genetic disease. Nat Rev Genet.

[R7] Maynard S, Schurman SH, Harboe C, de Souza-Pinto NC, Bohr VA (2009). Base excision repair of oxidative DNA damage and association with cancer and aging. Carcinogenesis.

[R8] David SS, O'Shea VL, Kundu S (2007). Base-excision repair of oxidative DNA damage. Nature.

[R9] Berquist BR, Wilson DM (2012). Pathways for repairing and tolerating the spectrum of oxidative DNA lesions. Cancer letters.

[R10] Dizdaroglu M (2005). Base-excision repair of oxidative DNA damage by DNA glycosylases. Mutat Res.

[R11] Sampath H, McCullough AK, Lloyd RS (2012). Regulation of DNA glycosylases and their role in limiting disease. Free radical research.

[R12] Hegde ML, Hazra TK, Mitra S (2008). Early steps in the DNA base excision/single-strand interruption repair pathway in mammalian cells. Cell research.

[R13] Sung JS, Demple B (2006). Roles of base excision repair subpathways in correcting oxidized abasic sites in DNA. The FEBS journal.

[R14] Hazra TK, Izumi T, Boldogh I, Imhoff B, Kow YW, Jaruga P, Dizdaroglu M, Mitra S (2002). Identification and characterization of a human DNA glycosylase for repair of modified bases in oxidatively damaged DNA. Proc Natl Acad Sci U S A.

[R15] Dallosso AR, Dolwani S, Jones N, Jones S, Colley J, Maynard J, Idziaszczyk S, Humphreys V, Arnold J, Donaldson A, Eccles D, Ellis A, Evans DG (2008). Inherited predisposition to colorectal adenomas caused by multiple rare alleles of MUTYH but not OGG1, NUDT1, NTH1 or NEIL 1, 2 or 3. Gut.

[R16] Shinmura K, Tao H, Goto M, Igarashi H, Taniguchi T, Maekawa M, Takezaki T, Sugimura H (2004). Inactivating mutations of the human base excision repair gene NEIL1 in gastric cancer. Carcinogenesis.

[R17] Vartanian V, Lowell B, Minko IG, Wood TG, Ceci JD, George S, Ballinger SW, Corless CL, McCullough AK, Lloyd RS (2006). The metabolic syndrome resulting from a knockout of the NEIL1 DNA glycosylase. Proc Natl Acad Sci U S A.

[R18] Sampath H, Batra AK, Vartanian V, Carmical JR, Prusak D, King IB, Lowell B, Earley LF, Wood TG, Marks DL, McCullough AK, L RS (2011). Variable penetrance of metabolic phenotypes and development of high-fat diet-induced adiposity in NEIL1-deficient mice. American journal of physiology.

[R19] Das A, Boldogh I, Lee JW, Harrigan JA, Hegde ML, Piotrowski J, de Souza Pinto N, Ramos W, Greenberg MM, Hazra TK, Mitra S, Bohr VA (2007). The human Werner syndrome protein stimulates repair of oxidative DNA base damage by the DNA glycosylase NEIL1. J Biol Chem.

[R20] Dou H, Theriot CA, Das A, Hegde ML, Matsumoto Y, Boldogh I, Hazra TK, Bhakat KK, Mitra S (2008). Interaction of the human DNA glycosylase NEIL1 with proliferating cell nuclear antigen. The potential for replication-associated repair of oxidized bases in mammalian genomes. J Biol Chem.

[R21] Hegde ML, Theriot CA, Das A, Hegde PM, Guo Z, Gary RK, Hazra TK, Shen B, Mitra S (2008). Physical and functional interaction between human oxidized base-specific DNA glycosylase NEIL1 and flap endonuclease 1. J Biol Chem.

[R22] Guan X, Bai H, Shi G, Theriot CA, Hazra TK, Mitra S, Lu AL (2007). The human checkpoint sensor Rad9-Rad1-Hus1 interacts with and stimulates NEIL1 glycosylase. Nucleic Acids Res.

[R23] Theriot CA, Hegde ML, Hazra TK, Mitra S (2010). RPA physically interacts with the human DNA glycosylase NEIL1 to regulate excision of oxidative DNA base damage in primer-template structures. DNA Repair (Amst).

[R24] Wiederhold L, Leppard JB, Kedar P, Karimi-Busheri F, Rasouli-Nia A, Weinfeld M, Tomkinson AE, Izumi T, Prasad R, Wilson SH, Mitra S, Hazra TK (2004). AP endonuclease-independent DNA base excision repair in human cells. Molecular cell.

[R25] Noren Hooten N, Kompaniez K, Barnes J, Lohani A, Evans MK (2011). Poly(ADP-ribose) polymerase 1 (PARP-1) binds to 8-oxoguanine-DNA glycosylase (OGG1). J Biol Chem.

[R26] Burkle A (2006). DNA repair and PARP in aging. Free radical research.

[R27] Luo X, Kraus WL (2012). On PAR with PARP: cellular stress signaling through poly(ADP-ribose) and PARP-1. Genes & development.

[R28] Schreiber V, Dantzer F, Ame JC, de Murci (2006). G. Poly(ADP-ribose): novel functions for an old molecule. Nature reviews.

[R29] Mangerich A, Herbach N, Hanf B, Fischbach A, Popp O, Moreno-Villanueva M, Bruns OT, Burkle A (2012). Inflammatory and age-related pathologies in mice with ectopic expression of human PARP-1. Mechanisms of ageing and development.

[R30] Chevanne M, Calia C, Zampieri M, Cecchinelli B, Caldini R, Monti D, Bucci L, Franceschi C, Caiafa P (2007). Oxidative DNA damage repair and parp 1 and parp 2 expression in Epstein-Barr virus-immortalized B lymphocyte cells from young subjects, old subjects, and centenarians. Rejuvenation research.

[R31] Muiras ML, Muller M, Schachter F, Burkle A (1998). Increased poly(ADP-ribose) polymerase activity in lymphoblastoid cell lines from centenarians. J Mol Med (Berl).

[R32] Piskunova TS, Yurova MN, Ovsyannikov AI, Semenchenko AV, Zabezhinski MA, Popovich IG, Wang ZQ, Anisimov VN (2008). Deficiency in Poly(ADP-ribose) Polymerase-1 (PARP-1) Accelerates Aging and Spontaneous Carcinogenesis in Mice. Current gerontology and geriatrics research.

[R33] Dizdaroglu M (2003). Substrate specificities and excision kinetics of DNA glycosylases involved in base-excision repair of oxidative DNA damage. Mutat Res.

[R34] Hu J, de Souza-Pinto NC, Haraguchi K, Hogue BA, Jaruga P, Greenberg MM, Dizdaroglu M, Bohr VA (2005). Repair of formamidopyrimidines in DNA involves different glycosylases: role of the OGG1, NTH1, and NEIL1 enzymes. J Biol Chem.

[R35] Ame JC, Hakme A, Quenet D, Fouquerel E, Dantzer F, Schreiber V (2009). Detection of the Nuclear Poly(ADP-ribose)-Metabolizing Enzymes and Activities in Response to DNA Damage. Methods in molecular biology (Clifton, NJ.

[R36] Bohr VA, Ottersen OP, Tonjum T (2007). Genome instability and DNA repair in brain, ageing and neurological disease. Neuroscience.

[R37] Gorbunova V, Seluanov A, Mao Z, Hine C (2007). Changes in DNA repair during aging. Nucleic Acids Res.

[R38] Lombard DB, Chua KF, Mostoslavsky R, Franco S, Gostissa M, Alt FW (2005). DNA repair, genome stability, and aging. Cell.

[R39] Grube K, Burkle A (1992). Poly(ADP-ribose) polymerase activity in mononuclear leukocytes of 13 mammalian species correlates with species-specific life span. Proc Natl Acad Sci U S A.

[R40] Hegde ML, Hazra TK, Mitra S (2010). Functions of disordered regions in mammalian early base excision repair proteins. Cell Mol Life Sci.

[R41] Wilson DM, Kim D (2011). Berquist BR and Sigurdson AJ. Variation in base excision repair capacity. Mutat Res.

[R42] Bouchard VJ, Rouleau M, Poirier GG (2003). PARP-1, a determinant of cell survival in response to DNA damage. Experimental hematology.

[R43] El-Khamisy SF, Masutani M, Suzuki H, Caldecott KW (2003). A requirement for PARP-1 for the assembly or stability of XRCC1 nuclear foci at sites of oxidative DNA damage. Nucleic Acids Res.

[R44] Bruner SD, Norman DP, Verdine GL (2000). Structural basis for recognition and repair of the endogenous mutagen 8-oxoguanine in DNA. Nature.

[R45] Doublie S, Bandaru V, Bond JP, Wallace SS (2004). The crystal structure of human endonuclease VIII-like 1 (NEIL1) reveals a zincless finger motif required for glycosylase activity. Proc Natl Acad Sci U S A.

[R46] Masson M, Niedergang C, Schreiber V, Muller S, Menissier-de Murcia J, de Murcia G (1998). XRCC1 is specifically associated with poly(ADP-ribose) polymerase and negatively regulates its activity following DNA damage. Mol Cell Biol.

[R47] Huber A, Bai P, de Murcia JM, de Murcia G (2004). PARP-1, PARP-2 and ATM in the DNA damage response: functional synergy in mouse development. DNA Repair (Amst).

[R48] Dou H, Mitra S, Hazra TK (2003). Repair of oxidized bases in DNA bubble structures by human DNA glycosylases NEIL1 and NEIL2. J Biol Chem.

[R49] Hanssen-Bauer A, Solvang-Garten K, Sundheim O, Pena-Diaz J, Andersen S, Slupphaug G, Krokan HE, Wilson DM, Akbari M, Otterlei M (2011). XRCC1 coordinates disparate responses and multiprotein repair complexes depending on the nature and context of the DNA damage. Environ Mol Mutagen.

[R50] Rouleau M, Patel A, Hendzel MJ, Kaufmann SH, Poirier GG (2010). PARP inhibition: PARP1 and beyond. Nat Rev Cancer.

[R51] Zaremba T, Curtin NJ (2007). PARP inhibitor development for systemic cancer targeting. Anti-cancer agents in medicinal chemistry.

[R52] Noren NK, Liu BP, Burridge K, Kreft B (2000). p120 catenin regulates the actin cytoskeleton via Rho family GTPases. The Journal of cell biology.

[R53] Nyaga SG, Lohani A, Evans MK (2008). Deficient repair of 8-hydroxyguanine in the BxPC-3 pancreatic cancer cell line. Biochem Biophys Res Commun.

